# Testicular Degeneration and Infertility following Arbovirus Infection

**DOI:** 10.1128/JVI.01131-18

**Published:** 2018-09-12

**Authors:** Giantonella Puggioni, Davide Pintus, Eleonora Melzi, Giorgio Meloni, Angela Maria Rocchigiani, Caterina Maestrale, Daniela Manunta, Giovanni Savini, Maria Dattena, Annalisa Oggiano, Massimo Palmarini, Ciriaco Ligios

**Affiliations:** a Istituto Zooprofilattico Sperimentale della Sardegna, Sassari, Italy; b MRC–University of Glasgow Centre for Virus Research, Glasgow, United Kingdom; c Istituto Zooprofilattico Sperimentale dell'Abruzzo e del Molise, OIE Reference Laboratory for BTV, Teramo, Italy; d AGRIS, Dipartimento per la Ricerca nelle Produzioni Animali, Olmedo, Italy; Instituto de Biotecnologia/UNAM

**Keywords:** arbovirus, bluetongue virus, infertility, sheep

## Abstract

Arboviruses can cause a variety of clinical signs, including febrile illness, arthritis, encephalitis, and hemorrhagic fever. The recent Zika epidemic highlighted the possibility that arboviruses may also negatively affect the male reproductive tract. In this study, we focused on bluetongue virus (BTV), the causative agent of bluetongue and one of the major arboviruses of ruminants. We show that rams that recovered from bluetongue displayed signs of testicular degeneration and azoospermia up to 100 days after the initial infection. Importantly, testicular degeneration was induced in rams experimentally infected with either a high (BTV-1_IT2006_)- or a low (BTV-1_IT2013_)-virulence strain of BTV. Rams infected with the low-virulence BTV strain displayed testicular lesions in the absence of other major clinical signs. Testicular lesions in BTV-infected rams were due to viral replication in the endothelial cells of the peritubular areas of the testes, resulting in stimulation of a type I interferon response, reduction of testosterone biosynthesis by Leydig cells and destruction of Sertoli cells and the blood-testis barrier in more severe cases. Hence, BTV induces testicular degeneration and disruption of spermatogenesis by replicating solely in the endothelial cells of the peritubular areas unlike other gonadotropic viruses. This study shows that a naturally occurring arboviral disease can cause testicular degeneration and affect male fertility at least temporarily.

**IMPORTANCE** During the recent Zika epidemic, it has become apparent that arboviruses could potentially cause reproductive health problems in male patients. Little is known regarding the effects that arboviruses have on the male reproductive tract. Here, we studied bluetongue virus (BTV), an arbovirus of ruminants, and its effects on the testes of rams. We show that BTV was able to induce testicular degeneration in naturally and experimentally infected rams. Testicular degeneration was caused by BTV replication in the endothelial cells of the peritubular area surrounding the seminiferous tubules (the functional unit of the testes) and was associated with a localized type I interferon response, destruction of the cells supporting the developing germinal cells (Sertoli cells), and reduction of testosterone synthesis. As a result of BTV infection, rams became azoospermic. This study highlights that problems in the male reproductive tract caused by arboviruses could be more common than previously thought.

## Abstract (precis)

During the recent Zika epidemic, it has become apparent that arboviruses could potentially cause reproductive health problems in male patients. Little is known regarding the effects that arboviruses have on the male reproductive tract. Here, we studied bluetongue virus (BTV), an arbovirus of ruminants, and its effects on the testes of rams. We show that BTV was able to induce testicular degeneration in naturally and experimentally infected rams. Testicular degeneration was caused by BTV replication in the endothelial cells of the peritubular area surrounding the seminiferous tubules (the functional unit of the testes) and was associated with a localized type I interferon response, destruction of the cells supporting the developing germinal cells (Sertoli cells), and reduction of testosterone synthesis. As a result of BTV infection, rams became azoospermic. This study highlights that problems in the male reproductive tract caused by arboviruses could be more common than previously thought.

## INTRODUCTION

The dramatic increase of both international travel and trade of goods and animals, in addition to deforestation and other ecological and climate changes, has created the conditions for the geographical expansions of several arthropod-borne viruses (arboviruses) (1, 2). Indeed, the majority of emerging and reemerging viruses affecting public health in the last 2 decades are transmitted by arthropods and diseases such as dengue, chikungunya, and West Nile encephalitis are no longer confined to the tropical areas of the globe (2–5).

Arboviruses can induce a variety of clinical outcomes in infected individuals, including febrile illness, encephalitis, arthritis, hemorrhagic fever, and even death (6). Importantly, arboviruses can also affect reproductive health. Many animal arboviruses, such as Schmallenberg virus, Akabane virus, Cache valley virus, and the human arbovirus Zika virus (ZIKV) are well known to be able to cross the placenta and to induce abortion or fetal malformations (7–15). Conversely, very limited information is available on the effects of arboviruses on the male reproductive tract. ZIKV has been detected in the seminal fluid of infected men, and sexual transmission of the virus has been confirmed in some cases (16). The potential presence of ZIKV in the male reproductive tract raises several questions of public health concern, including the possibility that arboviruses may affect male fertility. Importantly, these problems may pass unnoticed in infected patients with only mild clinical signs.

Arboviral infections of livestock can provide unique insights into the understanding of the basic mechanisms of arbovirus-host interactions. In these models, the hypotheses based on observations made in the field during the naturally occurring disease can subsequently be tested experimentally in the same animal species.

Bluetongue virus (BTV) is an arbovirus transmitted by Culicoides spp. and is the cause of bluetongue, one of the major arboviral diseases of ruminants (17–19). BTV induces a variety of clinical signs in infected sheep that range from subclinical infections to lethal hemorrhagic fever (20, 21). The clinical signs of bluetongue are primarily the result of damage of the capillary vessels which increases vascular permeability, resulting in edema and hemorrhages of the mucous membranes (21). Interestingly, studies conducted in the 1990s reported the presence of BTV in the semen of rams and bulls during the viremic phase, although the virus was considered to be detectable only in contaminating blood cells (22). Acute BTV infection in sexually mature rams has also been associated with transient infertility (22). In addition, studies conducted in Northern Europe during the 2006 BTV-8 epidemic showed that rams with naturally acquired bluetongue had lower semen quality, especially immediately after infection (23). Impaired semen quality was also observed in BTV-8-infected bulls (24). In addition, testicular lesions have been suggested to be a sequela of epizootic hemorrhagic disease virus infection (EHDV) in mule deer (25). EHDV is an arbovirus phylogenetically related to BTV and infecting deer (26).

In this study, we used rams naturally or experimentally infected with BTV to study the consequences of an arbovirus infection in the male reproductive tract. Our results indicate that a naturally occurring arbovirus of livestock causes testicular degeneration and azoospermia following pathogenetic mechanisms distinct from those described in other gonadotropic viruses. This study advances our understanding of the pathogenesis of arboviral diseases and suggests that testicular problems caused by arboviruses may be more common than previously appreciated.

## RESULTS

### Testicular degeneration in rams naturally infected by BTV-1_IT2013_.

**FIG 1:**
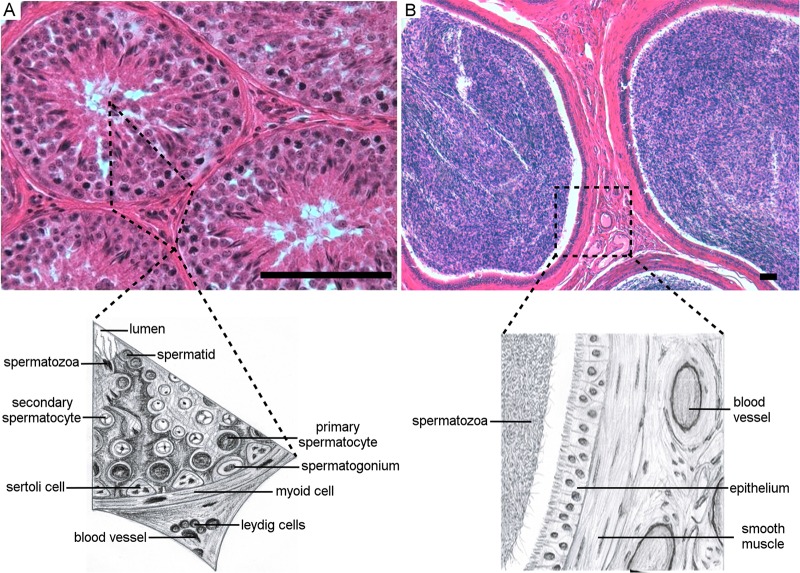
Histological features of the fertile ram testis and epididymis. (A) The top panel shows a micrograph of a section of the testis of a healthy ram stained with hematoxylin and eosin from one of the control mock-infected rams in this study. The bottom panel shows a drawing of a relevant portion of the micrograph indicated by the broken line. The seminiferous tubules contain the germinal cells. The Sertoli cells line the basal membrane and provide support for the immature germinal cells at different stages of maturation (spermatogonia, primary and secondary spermatocytes, and the mature spermatids). Among the tubules (peritubular spaces), there are blood vessels, macrophages, and Leydig cells. (B) The top panel shows a micrograph of a section of the epididymis stained with hematoxylin and eosin. The epididymis was collected from the same ram shown in panel A. The bottom panel shows a drawing of a portion of the micrograph indicated by the broken line. The epididymis is a tubule, delimitated by a line of epithelial cells lined on smooth muscle tissue, used by the mature spermatozoa to reach the vas deferens. Among the tubules of the epididymis (interstitial space) there are blood vessels and few scattered mononuclear cells. Scale bars, 100 μm.

In 2013, BTV-1_IT2013_ caused an outbreak of bluetongue in Sardinia (Italy). Farmers reported numerous episodes of reduced libido in rams and a decrease in the pregnancy rate in the affected flocks, suggesting fertility problems associated with the outbreak. In order to assess the consequences of BTV infection in the male reproductive tract, we identified 12 rams with confirmed diagnosis of BTV infection and euthanized them at approximately 30, 60, 100, and 150 days after infection (*n* = 3 per time point). We estimated the time of infection based on the general observation that onset of clinical signs of bluetongue occurs approximately 7 days postinfection (dpi). In addition, we identified eight healthy age-matched control rams seronegative for BTV. All animals had fully recovered from the disease and were clinically healthy at the time of euthanasia. At postmortem examination, both BTV-infected and uninfected rams showed no macroscopic lesions in any of their organs. Histologically, the appearance of the testes of the healthy control rams was normal. The functional units of the testes are the seminiferous tubules where spermatogenesis occurs (27) (Fig. 1A). Within the tubules, the Sertoli cells line the basal membrane and provide anatomical and nutritive support for immature germinal cells at different stages of maturation (spermatogonia, primary and secondary spermatocytes, and the mature spermatids) (Fig. 1A) (28). Between the tubules (peritubular or intertubular spaces), there are blood vessels, macrophages, lymphocytes, and the Leydig cells, which are responsible for the secretion of testosterone (Fig. 1A) (29). The mature and motile spermatozoa are collected in the epididymis (Fig. 1B), a tube connecting the testis to the vas deferens, which in turn transports the sperm to the ejaculatory ducts (27).

**FIG 2:**
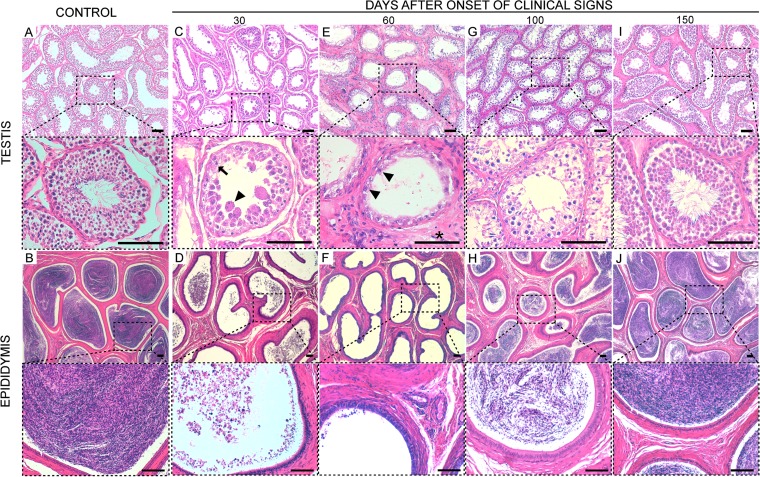
Testicular degeneration in rams naturally infected with BTV-1_IT2013_. Rams recovered from natural BTV-1_IT2013_ infection were sacrificed at 30, 60, 100, and 150 dpi. Postmortem, tissue sections from the testes and epididymis were stained with hematoxylin and eosin. Each panel is divided in a top and bottom micrograph. The bottom micrograph shows a larger magnification of the area delimited by the broken line in the top micrograph. (A) Representative micrographs of a section from the testis of a control ram. (B) Representative micrographs of a section from the epididymis of a control ram. (C) Representative micrographs of a section from the epididymis of ram killed at 30 dpi showing the lack of germinal cells in the tubules. In the bottom microphotograph, the arrowhead indicates a multinucleated giant cell, while the arrow indicates an apoptotic body. (D) Representative micrographs of a section from the epididymis of a ram killed at 30 dpi. Few degenerate spermatozoa are observed in the lumens of some tubules. (E) Representative micrographs of a section from the testis of a ram killed at 60 dpi. The space between the seminiferous tubules is thickened due to reactive fibrosis (asterisk). No germinal cells are observed in the tubules but only a line of Sertoli cells (arrowhead). (F) Representative micrographs of a section from the epididymis of a ram killed at 60 dpi. The lumens of the tubules contain no spermatozoa. (G) Representative micrographs of a section from the testis of a ram killed at 100 dpi. A multilayered germinative epithelium is observed in the tubules, suggesting that spermatogenesis is occurring, although not yet at normal levels. (H) Representative micrographs of a section from the epididymis of a ram killed at 100 dpi. Spermatozoa are evident in the lumens of the tubules but in relatively smaller numbers compared to those found in the same areas of control rams. (I) Representative microphotographs of a section from the testis of a ram killed at 150 dpi. No lesions are observed in the tubules, which show layers of germinal cells and normal spermatozoa. (J) Representative micrographs of a section from the epididymis of a ram killed at 150 dpi; the tubules appear normal with high concentrations of mature spermatozoa. Scale bars, 100 μm.

We observed in all six BTV-1_IT2013_-infected rams euthanized at 30 and 60 dpi different degrees of degenerative changes of the germinal epithelium of the testicular tubules compared to seronegative controls (Fig. 2). Although differences were found among individuals, in the majority of the cases most of the tubules displayed loss of germinal cells in the lumen, and the presence of apoptotic bodies and giant polynucleated cells resulting from arrested spermatogenesis (Fig. 2C). We could not distinguish mature spermatides in the testicular tubules, which were empty and lined with only a line of vacuolated Sertoli cells (Fig. 2C and E). The interstitial space was thickened as consequence of reactive fibrosis in some areas (Fig. 2E). In the epididymis of the same rams, tubules contained few degenerate spermatozoa (Fig. 2D) or appeared empty (Fig. 2F). In general, rams euthanized at 100 dpi showed a multilayered germinative epithelium and intact Sertoli cells with few spermatozoa in the majority of the epididymal tubules (Fig. 2G and H), indicating that spermatogenesis had started to be restored but was still affected. On the other hand, we detected no lesions in the testes of the rams at 150 dpi (Fig. 2I and J).

We also analyzed the semen (motility and percentage of live sperm) (30) and found a total absence of spermatozoa in the tail of the epididymis of rams up to 60 days postinfection, while values gradually returned to physiological levels between 100 and 150 dpi (Table 1).

**TABLE 1:** Motilities and percentages of live sperm in naturally BTV-1-infected rams^*a*^

Sperm parameter (%)	Controls	Naturally infected rams			
		30 dpi	60 dpi	100 dpi	150 dpi
Viability	80.2 ± 1	0	0	60.3 ± 0.6	79 ± 1.1
Motility	70.5 ± 0.8	0	0	42.6 ± 0.6	64.6 ± 0.6

a Values are expressed as means ± the standard errors of the mean.

### BTV induces testicular degeneration in experimentally infected rams.

**FIG 3:**
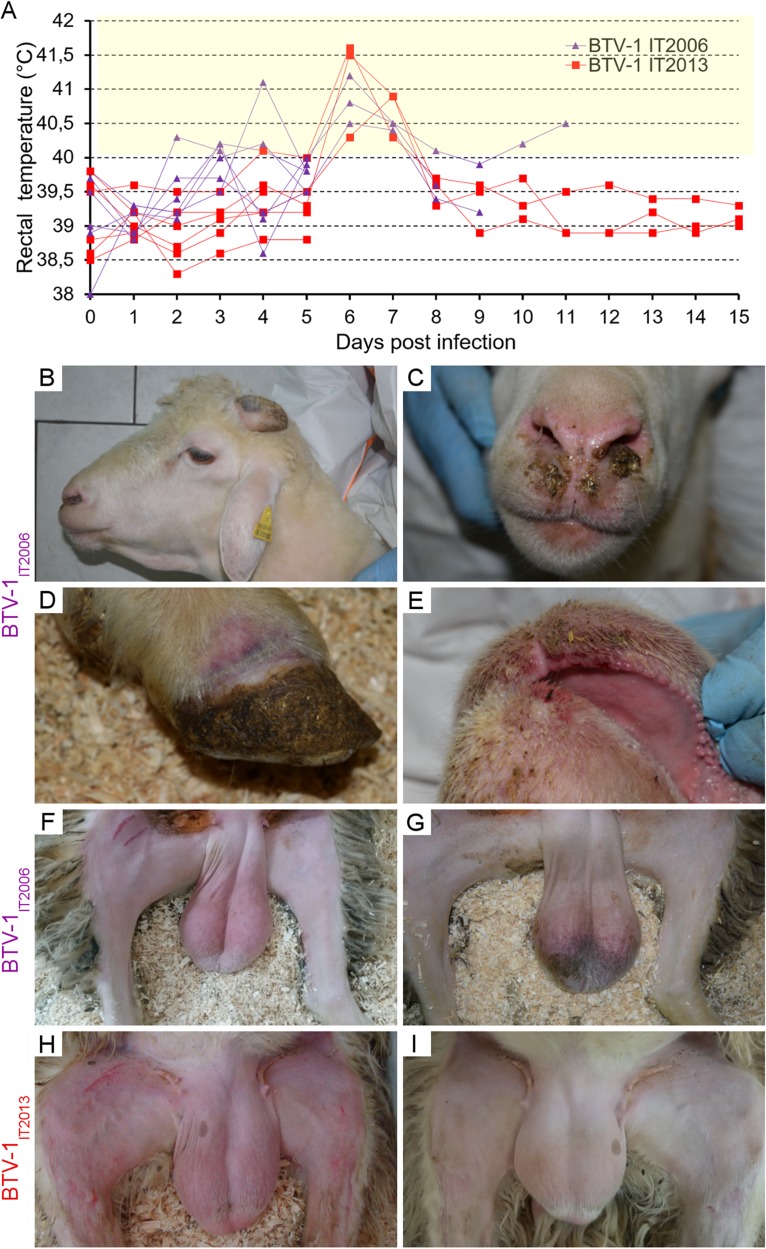
Rectal temperatures of rams infected with BTV-1_IT2006_ or BTV-1_IT2013_. (A) Graph representing the rectal temperatures of rams infected with BTV-1_IT2006_ (purple lines) or BTV-1_IT2013_ (red lines) throughout the duration of the experiment. Each line represents a single ram. (B to G) Representative images showing key clinical manifestations of rams infected with BTV-1_IT2006_ between 7 and 11 dpi, including intermandibular edema (B), nasal discharge (C), coronitis (D), erythema with ulcerations in the buccal mucosae (E), erythema of the scrotum (F), and cyanosis of the scrotum (G). No major lesions were observed in BTV-1_IT2013_-infected rams, with the exception of a mild erythema of the scrotum (H) visible in some rams at 7 dpi that resolved quickly (I).

In order to experimentally prove that BTV causes testicular degeneration, we infected 12 rams with either BTV-1_IT2013_ (*n* = 6) or with BTV-1_IT2006_ (another BTV-1 strain isolated in Sardinia in 2006) (*n* = 6). Four rams were used as mock-infected controls. All BTV-infected animals developed fever (Fig. 3A). A total of three rams for each group and two mock-infected controls were euthanized 5 dpi. The remaining rams were intended to be assessed at 15 dpi, but those infected with BTV-1_IT2006_ had to be euthanized at 8, 9, and 11 dpi since they developed severe clinical signs of bluetongue (Fig. 3), in accordance with the animal protocol used.

**FIG 4:**
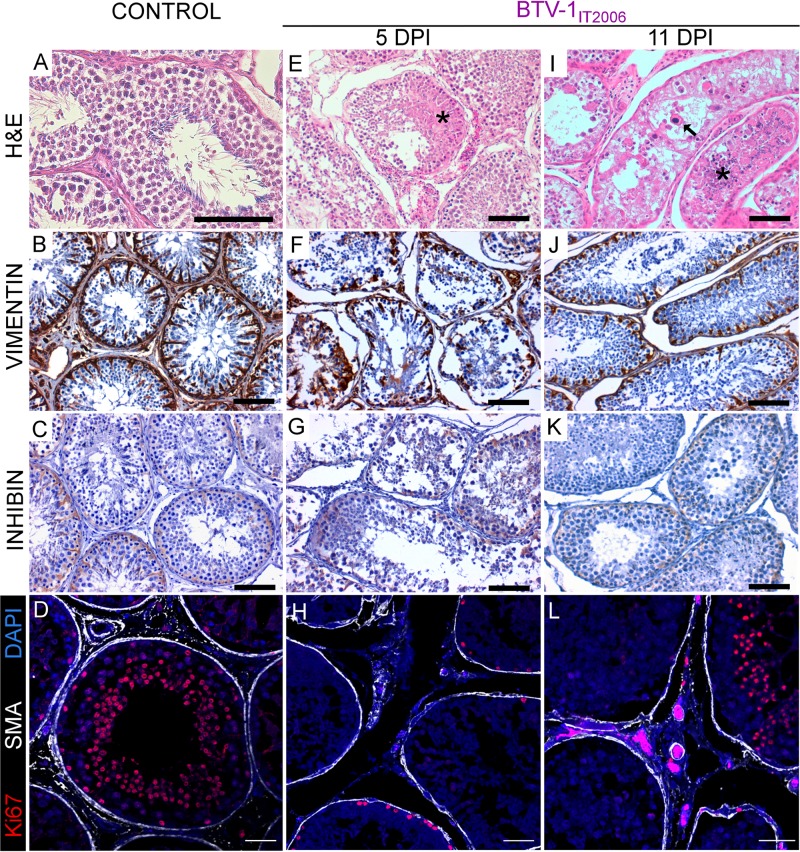
Testicular degeneration in rams experimentally infected with BTV-1_IT2006_. Panels on the left (A to D) show representative images from sections of a control ram testis. (A) Section stained with hematoxylin and eosin. (B) Section showing expression of vimentin. (C) Section showing expression of inhibin. (D) Confocal microscopy of the seminiferous tubules showing expression of Ki67 (in red) and of smooth muscle actin (SMA) (in white), as well as nuclear DAPI (4′,6′-diamidino-2-phenylindole) staining (in blue). Panels in the middle and on the right (E to L) show representative images from the testes of rams experimentally infected with BTV-1_IT2006_ and sacrificed at 5 (E to H) or 11 (I to L) dpi. (E) Micrograph of a section of the testis stained with hematoxylin and eosin. Degenerative changes of the germinal cells within the tubules with accumulation of serous eosinophilic material can be observed (asterisk). (F) Micrograph showing relative destruction of the layer of Sertoli cells in the tubules, as revealed by the detection of vimentin (showing in brown) by immunohistochemistry. There are relatively few Sertoli cells, which are often detached from the basal membrane. (G) Micrograph showing the relative lack of expression of inhibin by the Sertoli cells in an experimentally infected ram. (H) Confocal microscopy of a section of the testis. The seminiferous tubules show relatively infrequent expression of Ki67 (in red) by the germinal cells in division. SMA staining defining the tubules is shown in white, while DAPI staining is shown in blue. (I) Micrograph of a section of the testis stained with hematoxylin and eosin showing tubules severely damaged. Arrow points to germinal cells with nucleus of abnormal shape and dimension, while the asterisk indicates intratubular amorphous necrotic material. (J) Micrograph showing destruction of the layer of Sertoli cells in the tubules as revealed by the detection of vimentin (in brown) by immunohistochemistry. (K) Micrograph showing relative lack of expression of inhibin by the Sertoli cells in an experimentally infected ram. (L) Confocal microscopy as shown in panel D of a section of the testis of an experimentally infected ram. Scale bars: A, B, C, E, F, G, I, J, and K, 100 μm; D, H, and L, 50 μm.

At postmortem examination at 5 dpi, no gross-pathological lesions were evident in any of the experimentally infected or control rams. In BTV1_IT2006_-infected animals, we observed microvascular changes in the submucosae of the tongue, as well as in the superficial derma of the scrotal and facial skin, including vasculitis (data not shown). We also found vasculitis and inflammatory cells infiltration in the epididymis (data not shown). Importantly, in these rams we observed bilateral testicular degeneration, consisting of numerous tubules containing eosinophilic serous material and exfoliating germ cells with moderate edema and hyperemia in the interstitial spaces (Fig. 4A and E). Sertoli cells were lacking or exfoliating in the lumen of the tubules, as revealed by immunohistochemistry (Fig. 4F). Sertoli cells normally express high levels of vimentin (Fig. 4B) and low levels of inhibin (a nonsteroid hormone constitutively produced by these cells) (Fig. 4C) and are connected by tight junctions forming the so-called blood-testis barrier (31). We detected a very small number of germinal cells in active replication, as shown by Ki67 expression revealed by immunofluorescence (Fig. 4H). Interestingly, we observed no vasculitis in the peritubular spaces with only a few scattered CD3^+^ lymphocytes, which were also observed in the mock-infected controls (data not shown).

BTV-1_IT2006_-infected rams at late time points (8 to 11 dpi) showed cyanosis of the scrotal skin (Fig. 3G), while they histologically also displayed severe testicular degeneration with tubules containing eosinophilic amorphous necrotic material and germinal cells with abnormal nuclei (Fig. 4I). Sertoli cells were either absent or with abnormal morphology (Fig. 4J and K), and germinal cells expressing Ki67 were detected only in some tubules (Fig. 4L). No spermatozoa were observed in the lumens of the tubules in rams killed at 11 dpi. In semen extracted from the tail of the epididymis, both the number of live spermatozoa and their motility was normal in rams killed at 5 dpi, but values were significantly lower in rams killed at later time points (*P* < 0.0001; one-way analysis of variance [ANOVA]) (Table 2).

**TABLE 2:** Motilities and percentages of live sperm in experimentally BTV-1-infected rams^*a*^

Sperm parameter (%)	Controls	Experimentally infected rams			
		BTV_IT2006_		BTV_IT2013_	
		5 dpi	8–11 dpi	5 dpi	15 dpi
Viability	80.1 ± 0.9	80.3 ± 1.3	18.6 ± 1.3	80 ± 2.9	39 ± 1.1
Motility	70.2 ± 0.9	70 ± 1.1	9.6 ± 1.3	70.6 ± 2.3	23.6 ± 0.6

a Values are expressed as means ± the standard errors of the mean.

**FIG 5:**
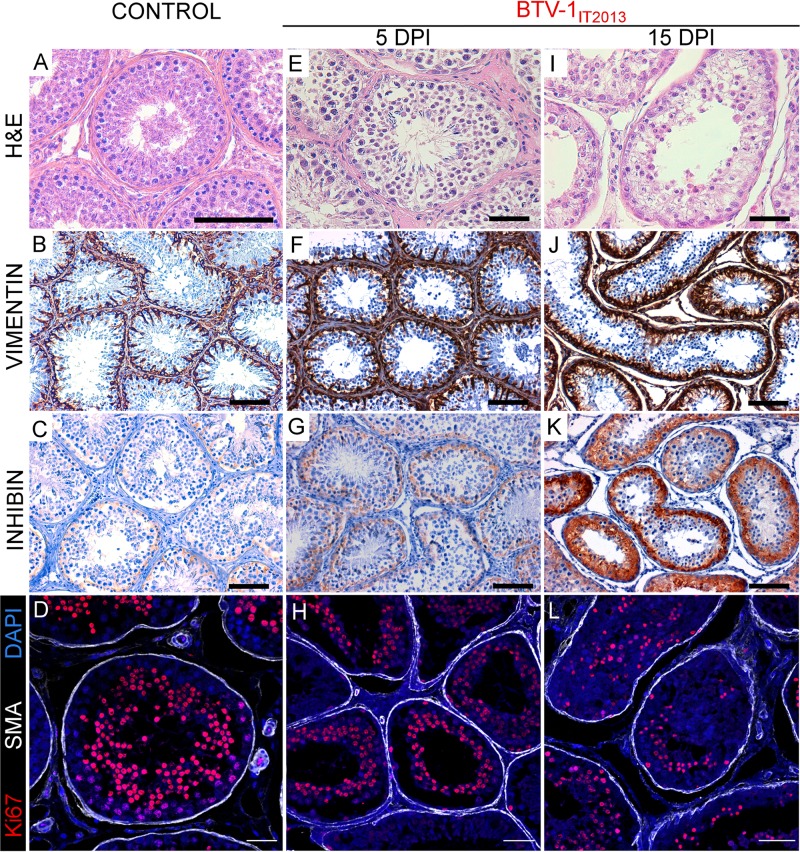
Experimental infection of rams with BTV-1_IT2013_ results in testicular degeneration. Panels on the left (A to D) show representative images from sections of a control ram testis. (A) Section stained with hematoxylin and eosin. (B) Section showing expression of vimentin. (C) Section showing expression of inhibin. (D) Confocal microscopy of the seminiferous tubules showing expression of Ki67 (in red) and of SMA (in white) as well as nuclear DAPI staining (in blue). The remaining panels (E to L) show representative images from the testes of rams experimentally infected with BTV-1_IT2013_ and sacrificed at 5 (E to H) or 15 (I to L) dpi. (E) Micrograph of a section of the testis stained with hematoxylin and eosin. The tissue appears morphologically normal and shows germinal cells at different stages of development in the tubules. (F) Micrograph showing an intact layer of Sertoli cells in the tubules as revealed by the detection of vimentin (showing in brown) by immunohistochemistry. (G) Micrograph as in panel B showing expression of inhibin by the Sertoli cells. (H) Confocal microscopy of a section of the testis. The seminiferous tubules show germinal cells in division as revealed by Ki67 (in red) expression. SMA staining defining the tubules is shown in white while DAPI in blue. (I) Micrograph of a section of the testis stained with hematoxylin and eosin showing tissue degeneration. A very limited number of germinal cells in the tubules are visible. (J) Micrograph showing an intact layer of Sertoli cells in the tubules as revealed by the detection of vimentin (showing in brown) by immunohistochemistry. (K) Micrograph showing upregulation of inhibin (in brown) expression by the Sertoli cells as determined by immunohistochemistry. Inhibin upregulation indicates functional activity of the Sertoli cells, while spermatogenesis is restored. (L) Confocal microscopy of a section of the testis. The seminiferous tubules show a reduced number of germinal cells in division (compare to panel D and H) as revealed by Ki67 expression (in red). SMA staining defining the tubules is shown in white while DAPI in blue. Scale bars: A, B, C, E, F, G, I, J, and K, 100 μm; D, H, and L, 50 μm.

In rams experimentally infected with BTV-1_IT2013_, we observed some microvascular changes in the superficial derma of the scrotal, epididymis, and facial skin (data not shown), but we did not detect any significant histological changes in the testes at 5 dpi (Fig. 5A to H).

Rams kept until 15 dpi showed no major clinical signs with the exception of moderate hyperthermia between 5 and 7 dpi (Fig. 3A) and erythema of the scrotum at 7 to 9 dpi (Fig. 3H and I). These rams also showed some degree of testicular degeneration, although in general the Sertoli cells remained intact (Fig. 5I and J), and the lesions were less severe compared to those induced by BTV-1_IT2006_, but no spermatozoa were present in the lumen (Fig. 5I). We could detect a robust expression of inhibin A and B (Fig. 5K) in the Sertoli cells of the infected rams compared to the control animals (Fig. 5C), suggesting an increasing functional activity of these cells normally following testicular dysfunction (32). We also observed a small number of Ki67^+^ cells in the lumens of the tubules (Fig. 5L).

The quality of the semen collected from the tail of the epididymis reflected the degenerative lesions observed in the testes (Table 2). As observed in rams infected with BTV-1_IT2006_, motility and viability of spermatozoa in rams infected with BTV-1_IT2013_ were comparable to those found in uninfected controls at 5 dpi but were significantly affected in animals sacrificed at 15 dpi (*P* < 0.0001; one-way ANOVA).

### Synthesis of testosterone is altered in experimentally infected rams.

**FIG 6:**
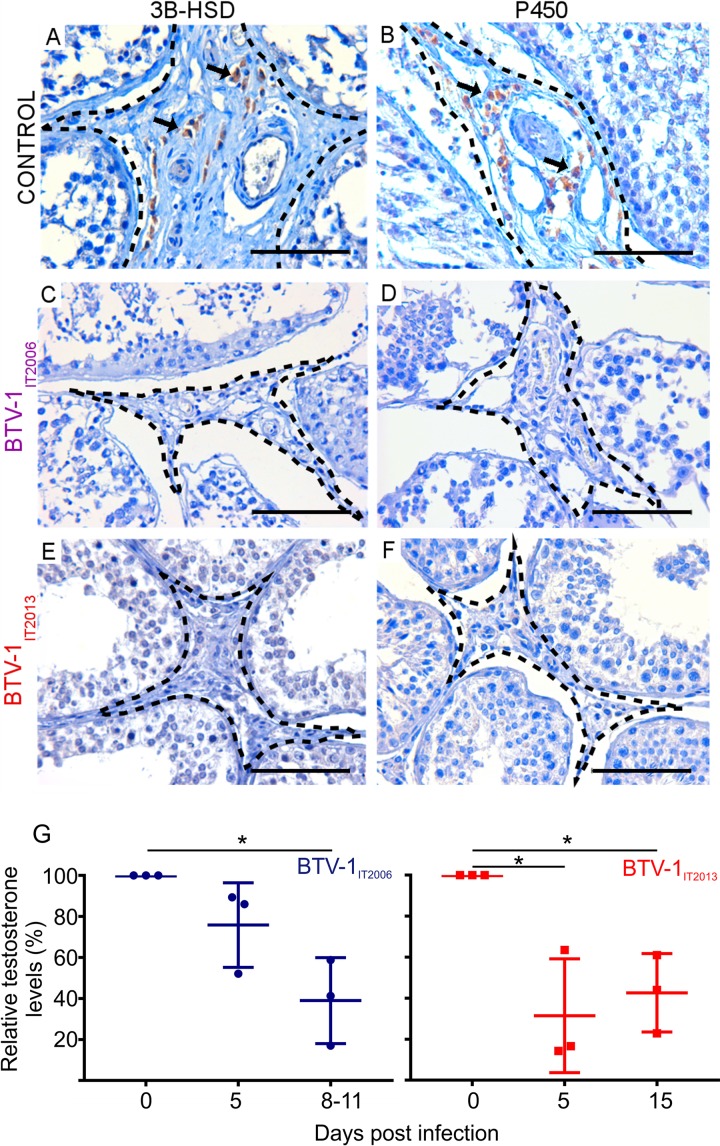
3β-HSD and P450 expression in BTV-1 experimentally infected rams and mock-infected controls. (A and B) Immunohistochemistry showing expression of 3β-HSD (A) and P450 (B) (in brown) by the Leydig cells in sections of the testes of mock-infected healthy control rams. Arrows point to Leydig cells. (C to F) Immunohistochemistry showing lack of expression of 3β-HSD (C and E) and P450 (D and F) by Leydig cells in the testes of rams infected with BTV-1_IT2006_ (C and D) or BTV-1_IT2013_ (E and F). The peritubular areas where the Leydig cells are located are highlighted with a broken line. Scale bars, 100 μm. (G) Graph representing the relative levels of testosterone, assessed by ELISA, in the blood of rams infected with either BTV-1_IT2006_ or BTV-1_IT2013_. Values are shown as percentages of the values of testosterone taken at day 0 before virus infection. Note the significant reduction in the levels of testosterone in rams infected with BTV-1_IT2006_ between 0 and 8 to 11 dpi (*P* < 0.05; one-way ANOVA) and in rams infected with BTV-1_IT2013_ between 0 and either 5 or 15 dpi (*P* < 0.05; one-way ANOVA).

Above, we described that rams infected either naturally or experimentally with BTV-1 displayed altered spermatogenesis. The Leydig cells in the testes have a fundamental role in maintaining spermatogenesis through testosterone biosynthesis (29). Here, we first evaluated by immunohistochemistry the functionality of Leydig cells by assessing the expression of 3β-hydroxysteroid dehydrogenase (3β-HSD) and cytochrome P450 aromatase (P450), two of the enzymes involved in testosterone production (33). Compared to healthy control rams (Fig. 6A and B), we found little or no immunoreactivity of either 3β-HSD or P450 enzymes in the interstitial space of the testes in both BTV-1_IT2006_-infected rams (at 5 and 11 dpi) and BTV-1_IT2013_-infected rams (at 5 and 15 dpi) (Fig. 6C to F). We also evaluated the levels of testosterone in the blood of all the inoculated animals by enzyme-linked immunosorbent assay (ELISA) (Fig. 6G). Under normal conditions, the levels of blood testosterone in rams varies considerably (34–36). Hence, we measured the levels of testosterone in each BTV-1-infected ram at day 5, and late at days 8 to 11 or day 15, relative to the levels found in the same rams at day 0 before infection (taken as 100). As expected, the data collected indicated that testosterone synthesis was impaired in all experimentally infected rams.

### BTV replicates in the testes of experimentally infected rams.

**FIG 7:**
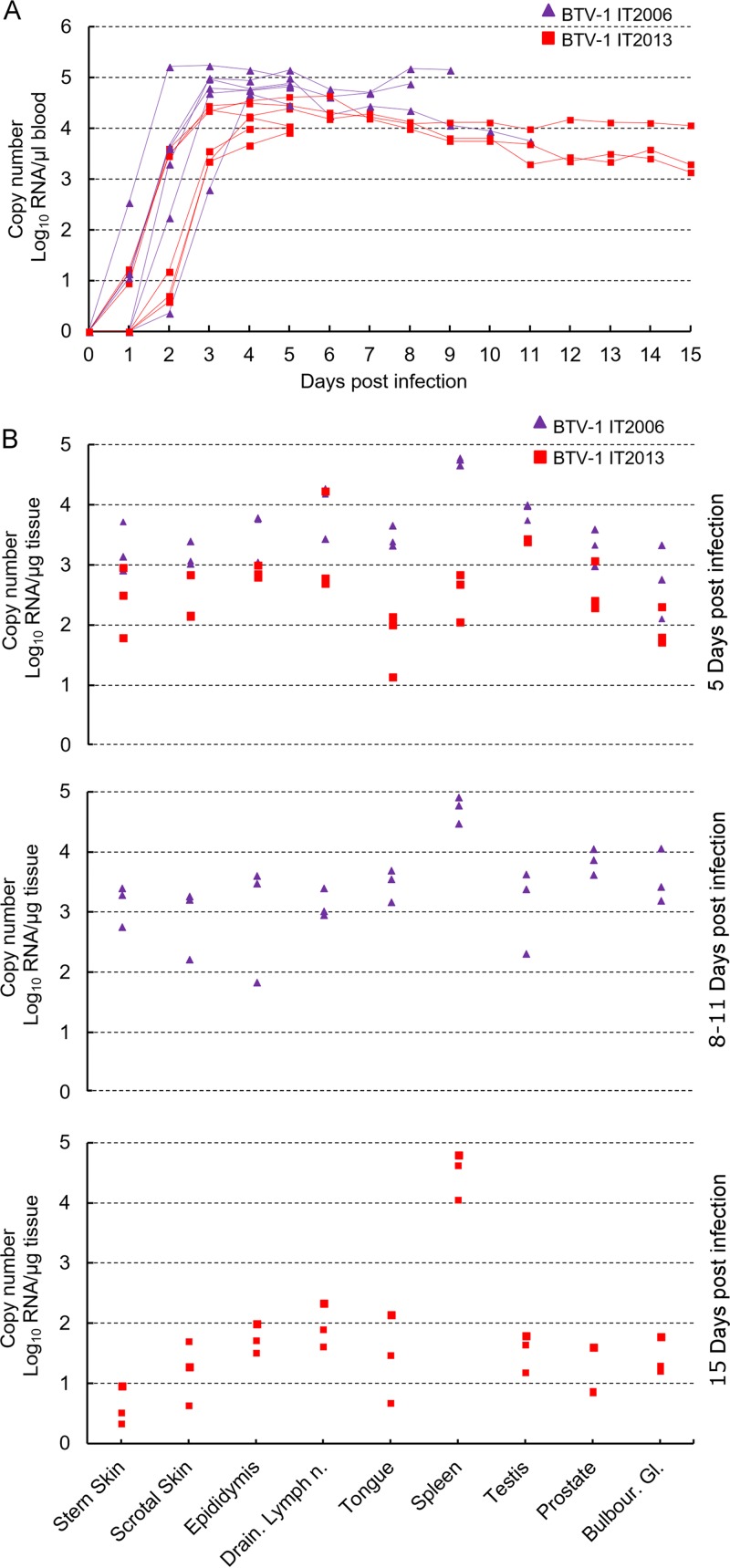
Detection of BTV RNA in blood and tissues of rams experimentally infected with BTV-1_IT20016_ or BTV-1_IT2013_. (A) Graph representing the relative levels of BTV-1 RNA by qRT-PCR in blood of experimentally infected rams. Each line represent the RNA amount of an individual ram infected with either BTV-1_IT2006_ (purple line) or BTV-1_IT2013_ (red line). (B) Graphs representing the relative levels of BTV-1 RNA by qRT-PCR in several organs of experimentally infected rams. Purple triangles represent rams infected with BTV-1_IT2006_, while red squares represent rams infected with BTV-1_IT2013_.

Next, we assessed whether testicular degeneration was induced as a result of BTV replication in the testes of infected animals. As expected, we detected viremia assessed by the quantification of BTV in the blood by quantitative reverse transcription-PCR (qRT-PCR) in all the experimentally infected rams throughout the duration of the study (Fig. 7A). BTV RNA was also quantified by qRT-PCR in RNA extracted from several organs, including the testes and epididymis, in all of the experimentally infected rams (Fig. 7B). Interestingly, the levels of viral RNA in the blood (at days 3, 4, 5, 7, and 8 dpi), as well as in other tissues (scrotal skin, tongue, spleen, and testis) of BTV-1_IT2006_ infected rams were significantly higher (*P* < 0.05, one-way ANOVA) compared to those in the equivalent tissues of BTV-1_IT2013_-infected animals. We isolated BTV-1_IT2006_ in cell culture from blood, spleen, testes, and tongue from all the experimentally infected rams sacrificed between days 5 and 11 postinfection. On the other hand, we were able to isolate BTV-1_IT2013_ only from the blood and spleens of infected animals at days 5 and 15 postinfection (data not shown). These differences are likely due to the significantly lower viral load of BTV-1_IT2013_ in tissues of infected rams compared to those infected with BTV-1_IT2006_.

**FIG 8:**
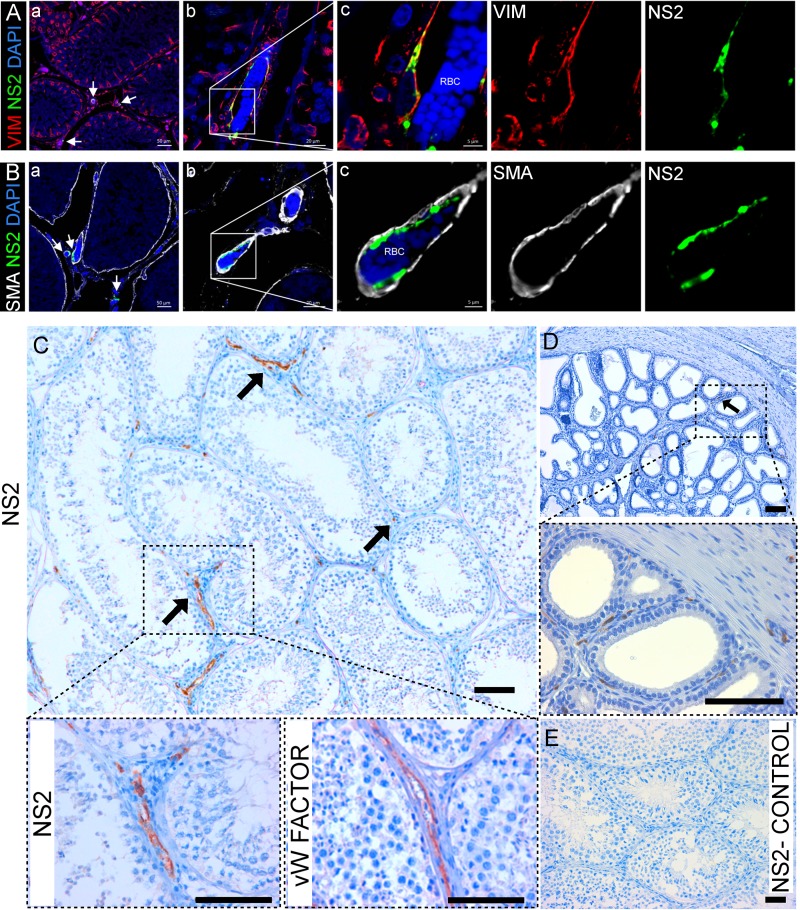
BTV-1 replicates in the endothelial cells of the peritubular vessels in the testes and in the prostate of experimentally infected rams. (A) Confocal microscopy of the testis of a ram infected with BTV-1_IT2006_ showing a section at low magnification (a) and at two progressively higher magnifications (insets, b and c) of a vessel localized in the intertubular space. BTV-1 NS2 is showed in green, vimentin in red, and DAPI in blue. (B) Confocal microscopy as in panel A showing pericytes (SMA+, in white) defining the vessel and surrounding the endothelial cells where BTV NS2 is detected (in green). RBC, red blood cells. (C) Representative image showing the localization of BTV NS2 protein (arrows) in the endothelial cells of the testis of a ram infected with BTV-1_IT2006_ (at 8 dpi) by immunohistochemistry. The area delineated with a dashed line is enlarged in the bottom panels where immunohistochemistry was carried out for the detection of NS2 or von Willebrand factor, a marker for endothelial cells. (D) Representative image showing the localization of BTV NS2 protein (arrow) in the endothelial cells of the blood vessels of the prostate of the same experimentally infected ram referred to in panel C. The area delineated with a dashed line is enlarged in the bottom panel where NS2 immunohistochemistry was also carried out. (E) No NS2 expression was detected by immunohistochemistry in the testis of a BTV-negative-control ram. Scale bars: A and B, insert a = 50 μm, insert b = 30 μm, and insert c = 5 μm; C, D, and E, 100 μm.

Next, we used immunohistochemistry and confocal microscopy to determine whether BTV actually replicated in the testes of experimentally infected rams. We detected the nonstructural protein 2 (NS2) of BTV in the endothelial cells of the smaller vessels of the peritubular spaces of the testes in both BTV-1_IT2006_- and BTV-1_IT2013_-infected rams at 5 dpi, as well as in the remaining rams infected with BTV-1_IT2006_ (euthanized at 8, 9, and 11 dpi) (Fig. 8A to C). By using antibodies against von Willebrand factor, vimentin, and smooth-muscle actin (SMA), we unequivocally identified the infected cells as endothelial cells (Fig. 8A to C) (37). We also detected BTV NS2 in the endothelial cells of the bulb-urethral glands and prostate (Fig. 8D). We did not detect BTV NS2 within the seminiferous tubules in any of the experimentally infected rams at either the early or late time points (Fig. 8E). In addition, we could not find any NS2-expressing cells in either the testes or in other tracts of the reproductive tracts of the remaining three rams infected with BTV-1_IT2013_ (and euthanized at 15 dpi). Similarly, we did not detect any BTV-infected cells by immunohistochemistry or confocal microscopy in the testes of the rams naturally infected with BTV_IT2013_ (data not shown).

### Antiviral responses in the testes of BTV-1 experimentally infected rams.

**FIG 9:**
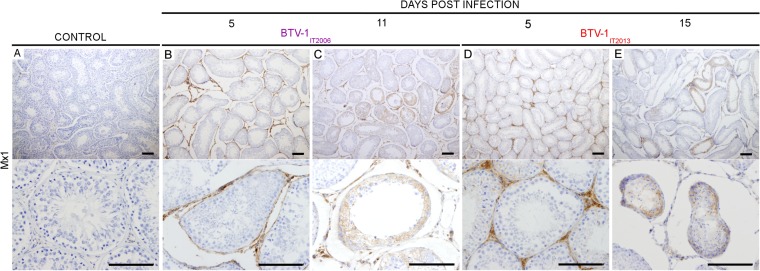
Type I IFN response in the testes of rams infected with either BTV-1_IT2006_ or BTV-1_IT2013_. (A) Representative image showing lack of detection, by immunohistochemistry, of Mx-1 in the testis of healthy mock-infected rams. (B to E) Mx1 expression is detected in the testes of rams infected with BTV-1_IT2006_ (B and C) and BTV-1_IT2013_ (D and E). At early time points (B and D), MX-1 is detected in the peritubular area, while at later time points it is also detected within a few tubules in either rams infected with BTV-1_IT2006_ or BTV-1_IT2013_. Scale bars, 100 μm.

**FIG 10:**
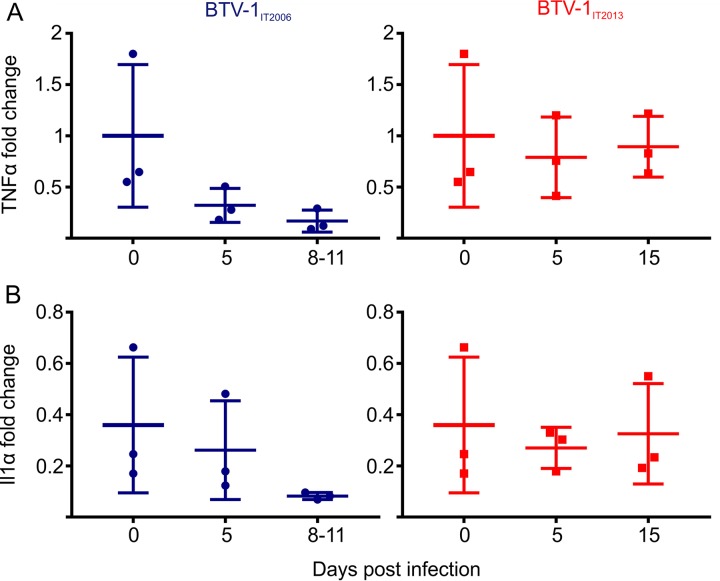
The proinflammatory cytokines TNF-α and IL-1α are not overexpressed in BTV-1 infected rams. (A and B) Graphs representing the relative levels of TNF-α (A) and IL-1α (B) in the testes of rams infected with BTV-1_IT2013_ or BTV-1_IT2006_. Values were normalized to the expression levels of the sheep 18S rRNA and are expressed as the relative fold change, taking as a unit the expression levels of one of the mock-infected controls. The sample values were normalized to housekeeping sheep 18S gene level. No statistically significant difference was noted in any of the groups tested (*P* > 0.05; one-way ANOVA).

The data described above suggested that BTV-1 replicates in the endothelial cells of the vessels of the ram reproductive tract. Previous studies have shown that BTV replication induces a type I interferon (IFN) response both *in vitro* and *in vivo* (38–43). Here, we assessed by immunohistochemistry the expression of Mx1, one of the core vertebrate IFN-stimulated genes (44), as indirect evidence of a host type I IFN response to BTV infection. As expected, we did not observe Mx1 expression in the testes of control rams (Fig. 9A). However, we observed strong Mx1 expression in the cells of the intertubular area in BTV infected rams (with both BTV_IT2006_ and BTV_IT2013_) at 5 dpi (Fig. 9B and D). We also observed Mx1 expression in the testes of rams euthanized at 11 dpi (BTV-1_IT2006_) and 15 dpi (BTV-1_IT2013_) (Fig. 9C and E). At these late time points, Mx1-positive cells were found both in the peritubular area and within few tubules. We found no differences in the levels of the proinflammatory cytokines tumor necrosis factor alpha (TNF-α) and interleukin-1α (IL-1α) in the testes of BTV-1-infected animals (at all time points) and healthy control rams by qRT-PCR (Fig. 10A and B).

## DISCUSSION

In this study, we showed that BTV, an arbovirus of ruminants, causes testicular degeneration and infertility, at least temporarily, in both naturally and experimentally infected rams. This is the first study demonstrating that an arbovirus causes testicular degeneration and affects spermatogenesis in the natural host of infection. We showed that testicular lesions induced by BTV are associated with (i) viral replication in the endothelial cells of the peritubular blood vessels of the testis, (ii) reduced production of testosterone in infected rams, and (iii) stimulation of a localized type I IFN response.

In infected rams, BTV replicates exclusively in the endothelial cells of the capillaries of the small vessels of the peritubular area of the testis. Hence, testicular degeneration due to BTV infection is likely the result of several overlapping factors. First, viral replication in the vessels is associated, at least temporarily, with a decrease in Leydig cell function. The Leydig cells are normally located in clusters among blood vessels and seminiferous tubules and are the primary source of testosterone in males (45). This hormone is released in the bloodstream, but it also diffuses locally (at higher concentrations than in the serum) to the seminiferous tubules, where it is essential for a variety of functions, including normal sperm production and release (46, 47), meiosis (48), Sertoli cell maturation (49), Sertoli cell-spermatid adhesion (50), and maintenance of the blood-testis barrier (49). The decrease in the Leydig cell function by itself can therefore have a dramatic effect on the physiology of the testis. It is important to note that the seminiferous tubules are completely avascular. Vessels are located in the peritubular area, and oxygen reaches the tubules and the sites of spermatogenesis exclusively by diffusion (51). Although the seminiferous tubules are normally hypoxic, proliferating germinal cells require high concentrations of oxygen (51). Testosterone is important for testicular microvascular blood flow. Therefore, decreased testosterone synthesis can affect the microcirculation of the testes and further augment pathology (52–55). In addition, it is possible that BTV replication in the endothelial cells of the testis by itself might also negatively affect the microcirculation in this organ. Furthermore, we showed that in infected rams BTV induces a robust localized type I IFN response. Interestingly, previous studies showed that intraperitoneal injections of IFN-α in rats affected spermatogenesis (56). Also, an excess of type I IFN in transgenic mouse models has been shown to affect spermatogenesis and eventually lead to sterility (57), likely as a result of impairment of the Sertoli cells, which have a functional type I IFN receptor and displayed a decrease in inhibin secretion. Hence, a sustained type I IFN response in the testes of BTV-infected rams may also contribute directly to pathology.

In BTV-infected rams, testicular degeneration did not seem to occur as a result of abundant inflammatory processes induced by virus replication. Indeed, we observed scarce or no inflammatory cell infiltration in the testes of BTV-infected rams, while we showed abundant T-cell infiltrates in their skin and tongues. We also found no overexpression of the proinflammatory cytokines TNF-α and IL-1α in the testes of BTV-infected rams. These observations are in line with the testes being an immune-privileged site where immune cell activation occurs less readily than in other organs (58, 59).

The reported sexual transmission of ZIKV (60) and the detection of this virus in the seminal fluid of infected patients (16) raised the possibility that a human arbovirus could cause male infertility. Indeed, different studies carried out in immunocompromised mouse models showed that ZIKV can damage the testes by infecting a variety of cells in this organ (61–63). ZIKV has been shown to replicate in Sertoli cells, resulting in their detachment from the basal membrane, destruction of the blood-testes barrier, and infiltration of inflammatory cells (62, 64). One study identified ZIKV replication in peritubular spindle-like myoid cells (α-smooth muscle actin positive) and in spermatogonia (61), while another found ZIKV to replicate in Leydig cells (63). Interestingly, Leydig cells have also been suggested to be a main site of replication for mumps virus, another gonadotropic virus (65, 66). Therefore, our study in BTV-infected rams strongly suggests an additional pathogenetic mechanism of virus-induced testicular degeneration resulting solely from virus replication in the endothelial cells of the testes. Interestingly, we observed testicular lesions both in rams with severe disease resulting from BTV-1_IT2006_ infection and in those displaying little or no clinical signs as a result of infection with BTV-1_IT2013_. In rams infected with the more virulent BTV-1_IT2006_, we observed complete destruction of the Sertoli cells and the blood-testis barrier. In these cases, virus-induced testicular lesions may be irreversible (27). Conversely, infection of rams with the less virulent BTV-1_IT2013_ induced less severe lesions that did not destroy the blood-testis barrier and are therefore likely reversible. Indeed, rams naturally infected with BTV-1_IT2013_ showed, at 150 dpi, no histopathological lesions, and their semen contained spermatozoa of correct morphology and motility.

BTV exists in nature in a variety of different serotypes and strains which have the tendency to evolve also by reassortment (67–70). Hence, the ability of BTV-1_IT2006_ and BTV-1_IT2013_ to induce testicular lesions may not be a property possessed by every BTV serotype/strain. However, replication of BTV in endothelial cells seems to be a common characteristic at least of the virulent strains of this virus (21). We speculate that BTV has the potential to induce testicular lesions in most cases, but the clinical significance, and therefore the opportunity to be diagnosed in the field, may vary.

Our study, in addition to the observations made during the recent Zika epidemic, raises the possibility that problems affecting the male reproductive tract may be an underappreciated consequence of possibly other arbovirus infections. For example, chikungunya virus has been found in the semen of a patient 30 days after the onset of clinical signs (71), but there are no data on the effects of this virus on male fertility. An additional confounding factor may be that most cases of arbovirus infections are subclinical or result in relatively mild symptomatology, and therefore they are not formally diagnosed. As discussed above, we also observed testicular lesions in rams infected with the low-virulence strain BTV-1_IT2013_ showing mild or no clinical signs of disease. Hence, arbovirus-induced infertility in men, especially if transient, may be undiagnosed in most cases.

Our study advances also the possibility that endotheliotropic human viruses may have underappreciated effects on male fertility. Ebola virus (EBOV), for example, has been found in the semen of infected men up to 565 dpi (72). The presence of EBOV in semen has been closely monitored in order to investigate sexual transmission in patients that have survived primary infection with this virus. However, we are not aware of studies investigating male fertility in Ebola survivors. Marburg virus, another endotheliotropic virus like EBOV, has also been found in the semen, and orchitis is one of the symptoms reported in some convalescent patients (73).

Bluetongue is a naturally occurring large animal model of arboviral diseases which has provided key insights into virus-host interactions (37). Large animals such as sheep allow the investigation of virus-induced pathology in the immunocompetent natural host and, furthermore, allows questions arising from observations of the natural disease to be addressed in an experimental setting using the same animal species. Virus pathogenesis is determined by virus replication in the face of host immunity, which in turn is fine-tuned by the long virus-host coevolutionary history. Hence, large animals and their arboviruses can provide additional perspectives to study viral pathogenesis.

## MATERIALS AND METHODS

### Ethical statement.

Animal experiments carried out in this study were approved by the ethical committee of the Istituto Zooprofilattico della Sardegna and further authorized by the Italian Ministry of Health (Ministero della Salute) in accordance with Council Directive 2010/63/EEC of the European Union and the Italian D.Igs 26/2014 (protocol 1248/2015-PR).

### Rams with naturally occurring BTV-1_IT2013_ infection.

A total of 12 rams with confirmed laboratory diagnosis of BTV infection from eight Sardinian flocks localized in the Northern part of the Island, were slaughtered (*n* = 3 per time point), with the agreement of the owners, at approximately 30, 60, 100, and 150 days after the onset of the clinical signs. Age-matched uninfected rams (*n* = 8 from two different flocks in the same geographical area) were used as healthy controls. At the necropsy, the testis and epididymis were adequately sampled from all these animals for histopathological examinations.

### *In vivo* experiments.

Two-year-old fertile rams (*n* = 12) were selected from flocks free from BTV infection. The presence of BTV in the blood was excluded by qRT-PCR (as described below), while the absence of antibodies toward the virus was assessed by competitive ELISA (74). In addition, testes of each ram were examined by echography to exclude preexisting pathologies. Semen was also collected from all the animals in order to establish that the numbers, morphology, and motility of spermatozoa were within the parameters of healthy animals. Rams were then separated into two groups and experimentally infected with BTV-infected blood according to an established protocol designed to reproduce clinical signs of the disease (75). Rams were infected intradermally (5 ml) and subcutaneously (3 ml) in an area in the proximal part of the neck with EDTA-infected blood derived from naturally sheep infected with either BTV-1_IT2006_ (a BTV strain circulating in Sardinia in 2006) or BTV-1_IT2013_ (a BTV strain circulating in Sardinia in 2013). Rectal temperature and clinical signs of infected and control rams were monitored daily for the duration of the whole experiment. A total of three rams of each group were euthanized at 5 dpi. The remaining rams infected with BTV-1_IT2013_ were euthanized 15 dpi, while those infected with BTV-1_IT2006_ were euthanized between days 8 and 11 because of the onset of severe clinical signs in accordance with the animal protocol used. A total of two rams for the 5-dpi time point and another two for the 15-dpi time point were used as mock-infected controls.

Blood samples were collected daily. In addition, during the postmortem examination, the following tissues were collected: testes, spleen, draining lymph nodes, epididymis, sternal skin, scrotal skin, prostate, bulbourethral, and tongue. Each sample was divided into halves, one of which was frozen at −80°C for qRT-PCR analysis, while the other was fixed in 10% neutral buffered formalin for histology, immunohistochemistry, immunofluorescence, and confocal microscopy.

### Semen analysis.

At necropsy, semen was collected directly from the tail of the epididymis from each ram included in this study. Sperm motility and vitality were assessed using the SCA (Sperm Class Analyzer) CASA System (Microptic) as suggested by the manufacturer.

### Histopathology, immunohistochemistry, and immunofluorescence.

Formalin-fixed tissues were embedded in paraffin according to routine laboratory protocols, cut into 4-μm-thick sections, and stained with hematoxylin and eosin. For immunohistochemistry and immunofluorescence, tissue sections were deparaffinized and rehydrated using routine procedures. Sections were incubated overnight at 4°C using primary antibodies specific against the following markers: BTV NS2 (38, 39), vimentin (Dako Agilent), von Willebrand factor (Dako Agilent), CD3 (Dako Agilent), MX-1 (76, 77), 3β-HSD (Santa Cruz Biotechnology), P450 Aromatase (AcrisAntibodies GmbH), inhibin α (Ventana Medical Systems), smooth muscle actin (Dako Agilent), and KI-67 (Dako Agilent). All of the antibodies were diluted in Antibody Diluents OP Quanto (Thermo Scientific) before use. Immunohistochemistry was carried out using Dako EnVision kit (Dako), and slides were counterstained with Mayer's hematoxylin. For immunofluorescence, secondary antibodies conjugated with fluorescent dyes were used (Alexa Fluor; Thermo Fisher Scientific). BTV NS2 was detected using the a tyramide signal amplification kit (Thermo Fisher Scientific) according to the manufacturer's protocol and as already described (37). Images were captured using Zeiss LSM710 confocal microscope and then analyzed by using Zen 2011 software (Zeiss).

### Virus isolation.

Virus isolation (78) was performed by passing sonicated blood once onto confluent monolayers of KC (a Culicoides sonorensis cell line) cells for 10 days at 28°C, followed by two blind passages on confluent monolayers of Vero (African green monkey) cells.

### qRT-PCR.

BTV-1 RNA in blood and other tissues was detected by qRT-PCR as previously described (79). Total RNA was extracted from blood using a QIAamp viral RNA minikit (Qiagen) or from tissues using the RNeasy fibrous tissue minikit (Qiagen) according to the manufacturer's instructions. Armored RNA (Asuragen) was used as an internal control to normalize for RNA extraction efficiency. Relative BTV genome copy numbers are expressed as log_10_/μl blood (or μg tissue) using a standard curve generated from the amplification of *in vitro*-transcribed segment 10 RNA. Blood and spleen samples from BTV-free sheep were used as controls.

Ovine TNF-α, IL-1α, and 18S rRNA were amplified by qRT-PCR from 50 ng of poly(A) RNA purified from the total testes RNA using an Oligotex mRNA kit (Qiagen, USA). Reverse transcription was carried out using a Quantitect reverse transcription kit (Qiagen). Sheep 18S rRNA was used as housekeeping gene. Data were analyzed using the Sequence Detection Systems 2.3 software (Life Technologies, USA), and the relative TNF-α and IL-1α gene expression levels were calculated using the 2^−ΔΔ*CT*^ method. Primer and probe sequences for all targets are available upon request.

### Testosterone quantification.

The concentration of testosterone in the sera of BTV-1_IT2006_- and BTV-1_IT2013_-infected rams was measured using a sheep testosterone ELISA kit (Shanghai Korain Biothec Co., China) according to the manufacturer's instructions.

### Statistical analysis.

Statistical analysis was performed using GraphPad Prism.
